# Understanding Drivers of Sustained Engagement in SMS‐Based Nutrition Programmes: A Realist Evaluation With an Equity Lens in Tanzania

**DOI:** 10.1111/mcn.70069

**Published:** 2025-07-29

**Authors:** Inka Barnett, Jessica Gordon, Deogardius Medardi, Mieke Snijder

**Affiliations:** ^1^ Institute of Development Studies (IDS) University of Sussex Brighton East Sussex UK; ^2^ Oxford Policy Management (OPM) Dar es Salaam Dar es Salaam Region Tanzania

**Keywords:** digital health, evaluation, health equity, low‐ and middle‐income countries, malnutrition, maternal‐child health services, programme

## Abstract

Child undernutrition remains a global challenge, intricately linked to systemic inequities in access to maternal and child health and nutrition (MCHN) services. While SMS‐based behaviour change communication (BCC) offers a scalable strategy to address this challenge, its effectiveness is often undermined by low and inconsistent user engagement. This study extends the impact evaluation of the mNutrition programme in Tanzania—a nationwide SMS‐based BCC programme aimed at improving maternal and child nutrition (MCN)—to investigate what drives sustained, long‐term engagement—an important but underexplored gap. The objective is to explore the mechanisms driving sustained engagement among mothers in rural Tanzania and how these are triggered by specific contextual factors and inequities. A realist approach was used, combining realist interviews with 40 sustained engagers (conducted across three sites between February and April 2019) and quantitative endline survey data. Retroductive analysis iteratively tested initial programme theories (IPTs) and derived Context‐Mechanism‐Outcome (CMO) configurations explaining sustained engagement. Findings showed sustained engagement resulted from the interplay between programme elements—such as content, SMS delivery (regularity, convenience, privacy), message tone (non‐judgmental, supportive), and perceptions of the sender—and inequity‐laden contextual realities, including time poverty, social isolation, undervaluation of women, gendered power dynamics within households and healthcare settings. The study concludes that digital BCC programmes must be context‐sensitive and equity‐oriented to achieve sustained engagement. Designing messages and delivery systems that reflect users' lived experiences and address structural vulnerabilities can enhance engagement and support more equitable nutrition outcomes.

## Summary

- Sustained engagement is not driven by any single feature of a digital BCC programme but by the interactions between programme elements (e.g., content, tone, delivery mode) and users' specific contexts and needs.
- Sustained engagement is dynamic and evolves over time. Digital BCC programmes should include mechanisms for ongoing feedback from users to adapt to their changing needs and contexts.
- Digital BCC programmes for MCN should aim to go beyond delivering behaviour change content by addressing broader inequities such as time poverty, social isolation, undervaluation, and gender imbalances within households to foster greater impact and sustained engagement.

## 1 Introduction

Child undernutrition—defined as inadequate intake of nutrients and/or recurrent infections leading to impaired growth and development—remains one of the most pressing global health challenges. It is deeply intertwined with systemic inequities in access to essential resources and services, including maternal and child health and nutrition (MCHN) services (Anarwat et al. 2021; Gube et al. 2024; Ijaiya et al. 2024; Karamagi et al. 2024; van Meurs et al. 2022). The magnitude of this issue is particularly severe in low‐ and middle‐income countries (LMICs), where barriers to accessing reliable health and nutrition services remain high. Digital delivery platforms have emerged as promising tools to expand the coverage of MCHN services, particularly in remote, underserved communities. Among these, mobile‐based behaviour change communication (BCC) interventions have gained prominence for their ability to disseminate information and promote healthier behaviours at scale (Janmohamed et al. 2020; Keats et al. 2021; Mildon and Sellen 2019; Zarnowiecki et al. 2020). SMS text messaging, in particular, has proven uniquely equitable and widely adopted due to its simplicity, affordability, scalability, and compatibility with basic mobile phones and 2G networks (Cunningham et al. 2024; Dobson et al. 2024; Jordan et al. 2025; Materia et al. 2023; Rothstein et al. 2021). Despite 25 years of digital health advancements, SMS remains a key medium for reaching poor and marginalised populations.(Dobson et al. 2024; Jordan et al. 2025; Materia et al. 2023; Rothstein et al. 2021). However, the impact of SMS‐based BCC programmes on maternal and child nutrition (MCN) outcomes remains inconsistent (Cunningham et al. 2024; Dao et al. 2023; Graziose et al. 2018; Islam et al. 2021; Kusyanti et al. 2022; Poorman et al. 2015). One major challenge is limited user engagement, which significantly undermines programme effectiveness (Dumas et al. 2021; Kusyanti et al. 2022; Rothstein et al. 2021). Engagement —conceptualised as an intermediate step linking message delivery to behavioural change—is a critical determinant of impact (Palsola et al. 2020). Yet, most existing studies focus on barriers to engagement, such as poor network connectivity, disparities in mobile phone ownership and access, and digital literacy (Currie et al. 2023; Kaihlanen et al. 2022; Rothstein et al. 2021). What remains underexplored are the motivating factors behind sustained engagement, defined as ongoing, active interaction with BCC content over time (Grady et al. 2023). This gap limits the ability to design SMS‐based BCC programmes that not only attract initial user attention but also maintain engagement long enough to yield meaningful behavioural changes. Addressing this gap, particularly through an equity lens, is essential for improving programme efficacy. This study shifts the discourse from barriers to engagement toward understanding what motivates sustained engagement in SMS‐based BCC interventions for MCN. By identifying the underlying reasons why some users consistently read and engage with SMS‐based BCC, this study offers practical insights for designing more impactful and equitable digital health programmes. Specifically, we examine emotional, social, and systemic drivers of sustained engagement among marginalised populations. The engagement‐outcome dynamic in digital BCC is complex and context‐dependent (Grady et al. 2023; Perski et al. 2017; Yardley et al. 2016). For intervention targeting discrete behaviour, short‐term engagement may be sufficient. However, when aiming to address a range of interconnected behaviours, as in MCN, sustained engagement becomes essential for meaningful change (Mello et al. 2019).

### 1.1 Programme

This study is nested within the impact evaluation of the mNutrition programme in Tanzania (hereafter mNutrition) (Barnett et al. 2020). mNutrition, a global SMS‐based initiative implemented between 2013 and 2020 across 12 countries in sub‐Saharan Africa and South Asia. Supported by the UK's Foreign, Commonwealth and Development Office (FCDO) and organised by the GSMA, the programme leveraged mobile network operators and third parties to deliver BCC. In Tanzania, the programme delivered MCN‐related SMS messages via the Wazazi Nipendeni (WN) mHealth platform.rm (Table 1). Example messages are available in Appendix 1.

**Table 1:** Key features of mNutrition implemented in Tanzania.

Features	Description
Delivery platform	‘Healthy Pregnancy, Healthy Baby’ (HPHB) SMS programme, also known as the Wazazi Nipendeni SMS programme.
Target group	Pregnant women and mothers/caregivers of children under 2 years.
Behaviour change messages	120 tailored SMS messages addressing maternal and child diet and micronutrient intake, breastfeeding, complementary feeding, and childcare. Messages were time‐sensitive, aligned with pregnancy stages or child age, reduced in frequency as children grew, developed by global experts and adapted to the local context, and based on formative research and pilot testing. Content followed Tanzania's National Nutrition Guidelines and was delivered in Swahili.
Coverage	National, available across all mobile networks, and provided free of charge.

The impact evaluation, conducted between October 2016 and August 2020, included a quantitative randomised controlled trial (RCT) with 90 treatment communities (*n* = 1428 mothers) and 90 control communities (*n* = 1407) alongside three in‐depth qualitative rounds in a subset of treatment communities. The evaluation found minimal programme impact on MCN practices and no effect on child nutritional status in rural Tanzania—partly attributed to low reach and engagement, with only 24% of participants reporting having read an SMS (Barnett et al. 2020).

This study extends the impact evaluation findings by focusing on ‘sustained engagers’, defined as mothers who self‐reported receiving and reading at least one mNutrition SMS per month for six consecutive months or more. Quantitative endline data revealed that only 172 of 1428 treatment mothers (12%) met this criterion. Using a realist evaluation approach this study aims to address the following questions: What mechanisms drive sustained engagement with the mNutrition programme among mothers in rural Tanzania, and how are these mechanisms triggered by the programme in relation to mothers' specific contexts and the inequities experienced within these contexts? This study is novel because it shifts the discourse from understanding barriers to exploring the deeper motivations behind sustained engagement with SMS‐based BCC programme. It offers a granular, equity‐centred analysis of why some rural Tanzanian mothers continue to engage with messages over time, using realist evaluation to uncover mechanisms within specific contextual inequities—thereby enhancing the design and impact of future digital nutrition programmes.

## 2 Methods

This study primarily draws on the third round of qualitative data collection from the mNutrition impact evaluation, complemented by selected indicators from the quantitative endline survey. The study was guided by realist evaluation principles. Realist evaluations posit that a programme introduces new resources into an existing social system, which, when interacting with specific contextual features of the recipient, may trigger internal reasoning (e.g., thought processes or emotional responses), resulting in specific outcomes, such as sustained engagement (Pawson and Tilley 1997). This interaction of resources and reasoning, known as a mechanism (M), explains how a programme works and why the same programme may yield different outcomes (O)—or fail—depending on the e unique contexts (C) in which individuals are situated (Dalkin et al. 2015; Pawson 2013).

The study adhered to Pawson and Tilley's (1997) realist evaluation framework and followed the RAMSESS II reporting standards (Wong et al. 2016):

### 2.1 Step 1: Development of the Initial Programme Theory (IPT)

Realist evaluations use Initial Programme Theories (IPTs) to hypothesise for whom, why, and in which context a programme may work, which are then tested and refined with empirical evidence. Five IPTs were developed based on existing literature and initial findings on mothers' engagement with mNutrition from the first two rounds of qualitative data collection for the evaluation (Barnett et al. 2019; Barnett et al. 2018) (see Appendix 2). The development of the IPTs was also informed by frameworks modelling factors associated with (sustained) engagement in digital health behaviour change programmes, including the Digital Health Engagement Model (DIEGO) (O'Connor et al. 2016), the NASSS framework (Greenhalgh et al. 2017), and the conceptualizations by Yardley et al. (2016) and Perski et al. (2017). The WHO's Social Determinants of Health (SDH) Framework (WHO 2010) was also consulted to address potential contextual and systemic factors and inequities impacting mothers' access to and use of conventional MCHN services.

### 2.2 Step 2: Study Site Selection

The IPTs were iteratively tested during the final round of qualitative data collection from February to April 2019. Using a multiple case study design, data were collected across three distinct sites: Mufindi 1, Mufindi 2, and Iringa Rural. These sites were purposively selected from the treatment arm of the broader RCT to ensure variation in contextual characteristics relevant to engagement with the mNutrition programme. Each site encompassed multiple neighbouring communities—six in both Mufindi 1 and Mufindi 2, and nine in Iringa Rural—because the number of sustained engagers within individual communities was limited. Including multiple communities per site allowed for a sufficient and diverse sample of participants while maintaining contextual coherence within each site. Site selection was guided by maximum variation sampling principles. Criteria included geographic remoteness (assessed using GPS mapping), distance to health facilities, and administrative diversity across the Iringa Region. This approach aimed to capture a broad range of experiences and contextual factors that could influence sustained engagement.

From the endline survey data, all mothers identified as sustained engagers—defined as those who self‐reported receiving and reading at least one SMS per month for six consecutive months—within the selected sites were invited to participate in in‐depth interviews (IDIs). A total of 43 eligible women were contacted, and 40 agreed to participate. The three who declined cited constraints related to work obligations or travel.

### 2.3 Step 3: Data Collection, Quality Control and Analysis

Participants participated in a single, approximately 60‐min face‐to‐face realist interview conducted in Swahili. A flexible, pilot‐tested semi‐structured topic guide was used to explore the IPTs, focusing specifically on mothers' specific contexts and the mechanisms hypothesised to motivate sustained engagement, while also allowing for the probing of unexpected themes (see Table 2, for example questions). To ensure data quality and methodological rigour, multiple strategies were implemented throughout the study. First, all interviews were conducted by experienced Tanzanian researchers who received targeted training in realist interviewing techniques, including theory gleaning and refining strategies. (Manzano 2016; Pawson and Tilley 1997). Interview guides were piloted and adapted iteratively to ensure clarity and contextual relevance. Daily debriefings and real‐time reflective sessions were held between field teams and study leads to assess the quality of data collection, ensure fidelity to the interview protocol, and incorporate emerging insights into subsequent interviews. Information saturation was assessed during these debriefings. Saturation was considered reached when interviews no longer yielded new information or variation relevant to the emerging CMO configurations across the three sites. All participants provided informed consent, and ethical approval was obtained from the Tanzania Food and Nutrition Centre (TFNC), the President's Office, regional and district authorities.

**Table 2:** Examples of topic guide questions aimed at testing IPTs.

*Mechanisms*
How do you feel when you receive an mNutrition SMS? Can you explain why you feel that way? Does it motivate you to continue to engage, why? (e.g., helps reduce loneliness, feel that your concerns are validated; feels special; feels monitored/observed). SMS with information on MCN can be accessed on your mobile phone at a time and place convenient for you and without talking to anybody (e.g., a health worker) which can make it easier for busy mothers. Was this the case for you? Does it motivate you to continue to engage, why? Were there ever short periods when you stopped engaging with mNutrition? What motivated you to engage again and to continue to engage?
*Mothers’ contexts*
How easy it is for you to get access to credible information on MCN? How much time do you have to look for information on child nutrition actively? Do you experience any challenges when trying to access information (e.g., in the health facility, lack of time). How much influence do you have on decisions regarding childcare in your household? Do you think the SMS's influenced your influence on these decisions? How and why? How supported do you feel as a new mother? Do you feel you can easily ask for help? Have the SMS's changed how you feel? Why?

All interviews were audio‐recorded, transcribed in Swahili, and translated into English by experienced bilingual researchers. A subset of transcripts was cross‐checked against the original recordings to ensure translation accuracy, with discrepancies resolved through discussion. A retroductive data analysis approach was employed, as recommended for realist evaluations (Gilmore et al. 2019). It combined deductive and inductive reasoning to iteratively test and refine IPTs, evolving them into specific Context‐Mechanism‐Outcome (CMO) configurations to explain sustained engagement with mNutrition (II 2018). Transcripts were initially coded deductively, using the IPTs as a framework to guide the coding of evidence into categories of context, mechanisms, and outcomes (Braun and Clarke 2006; Miles and Huberman 1994). Inductive reasoning was then applied to uncover new patterns or insights from the data that refined or challenged the IPTs, which were subsequently tested deductively against the entire data set to ensure validity. Empirically derived CMOs were generated from emerging patterns linking specific contexts and mechanisms to outcomes, with mechanisms disaggregated into resources and reasoning. The seven CMOs with the strongest empirical support in the data were grouped under their corresponding IPTs (see Table 3). Data coding and interpretation were conducted independently by two researchers (I.B. and J.G.) using NVivo 1.3, followed by triangulation and consensus‐building to enhance analytic validity. These steps collectively ensured that the findings were robust, contextually grounded, and reliably linked to the realist evaluation framework.

**Table 3:** Refined initial programme theories (IPTs) and seven specific context‐mechanism‐outcome configurations explaining sustained engagement with mNutrition.

		Mechanism			
IPT/CMO	Context	Programme resource	Mothers' reasoning		Outcome
IPT_1: In a context in which mothers are uncertain about correct MCN and have a desire for guidance, if they receive regular information thorough SMS, they are likely to persist to engage with the programme as it addresses their need for guidance.					
CMO1	High anxiety about child health, feeling helpless and without control.	Regular practical advice tailored to a child's age.	Feel more in control because of the consistent reassurance through the messages.		Sustained engagement to reduce anxiety and maintain sense of control.
IPT_2: In a context in which mothers feel socially isolated, if they receive regular tailored SMS, they will continue to engage as the messages trigger in them a feeling of social connection and support.					
CMO2	Feel unvalued and lacking acknowledgement for juggling childcare, household chores and farm work.	Emotional support.	Feeling of being ‘seen’ and acknowledged which builds self‐esteem and self‐belief that efforts are valued.		Sustained engagement to receive validation of mothering efforts.
CMO3	Feel socially isolated and abandoned with their new babies.	Regular social support.	Counteract feeling of loneliness by providing regular ‘check ins’ from the outside world, feeling of being part of a community of mothers in a similar situation.		Sustained engagement to combat feelings of loneliness.
IPT_3: In a context in which mothers have limited decision‐making power regarding childcare, if they receive SMS with credible information that empowers them to reshape intrahousehold negotiations regarding child feeding and care they will continue to engage.					
CMO4	Lack of resources and decision‐making power to improve MCN practices despite desire to do so.	Credible information from authorities.	Feeling empowered by written support for demands in intrahousehold negotiations.		Sustained engagement as provides valuable support in intra‐household negotiations.
IPT_4: In a context where mothers want MCN advice, but internal and/or external barriers impede their access to available sources, if they receive easily accessible information through SMS, they are likely to continue to engage as it alleviates stress associated with seeking information.					
CMO5	Feel guilty as busy daily schedules prevent regularly take up conventional MCN resources.	Easily accessible information	Feeling of guilt is alleviated through engagement with messages.		Sustained engagement to ease feelings of guilt.
CMO6	Feels intimidated by health workers and fear to be publicly berated or judged.	Anonymous information provided free of judgement.	Mothers feel supported without fear and encroachment on privacy.		Sustained engagement to receive advice without fear and pressure.
IPT_5: Beliefs held around the sender of the SMS (whether accurate or not) will influence mothers sustained engagement with the SMS programme as these beliefs evoke a range of emotions including the feeling of being monitored (e.g., by government authority).					
CMO7	Respect higher authorities and fear that failure to engage or comply results in (public) admonishment.	Regular check‐ups and monitoring.	Feeling of being closely observed.		Sustained engagement to avoid admonishment.

## 3 Results

### 3.1 Participant Characteristics

Demographic data for the qualitative participants, extracted from the quantitative endline survey, are detailed in Table 4. Mothers were on average 27 years old, with a wide age range (SD = 9.5). Most were less poor (*n* = 20), had two children (*n* = 23) or were first‐time mothers (*n* = 11), and were married (*n* = 33). The majority engaged with mNutrition for at least 12 months (*n* = 35), read all SMS messages (*n* = 30), and found the content ‘always’ useful (*n* = 21). Most (*n* = 24) reported implementing ‘all or most’ of the MCN advice. No structural barriers to accessing the programme were reported, as mothers either owned a mobile phone (*n* = 31) or had consistent access, could easily charge it, had minimal connectivity issues, and were able to read and understand the SMS content (Table 4).

**Table 4:** Demographics of qualitative participants and engagement with and responses to mNutrition, overall and by site.

		Total		Mufindi 1		Mufindi 2		Iringa rural	
		*N* = 40		*N* = 10		*N* = 11		*N* = 19	
Age (years), mean (SD)		27.4	9.5	26.8	4.9	29.1	6.7	31.3	5.7
Socioeconomic status, *n* (%)									
	Very poor	7	17.5	2	20.0	1	9.1	2	10.5
	Poor	13	32.5	2	20.0	2	18.2	8	42.1
	Less poor	20	50.0	6	60.00	8	72.7	9	47.4
Number of children (at endline)									
	One child	11	27.5	5	50.0	3	27.3	3	15.8
	Two children	23	57.5	3	30.0	7	63.6	13	68.4
	≥ Three children	6	15.0	2	20.0	1	9.1	3	15.8
Marital status, *n* (%)									
	Unmarried	7	17.5	3	30.0	2	18.2	2	10.5
	Married (mono‐ or polygamous)	33	82.5	7	70.0	9	81.8	17	89.5
Lengths of engagement with SMS programme (n, %)									
	Between 6 and 11 months	5	12.5	1	10.0	2	18.2	3	15.8
	Between 12 and 24 months	35	87.5	9	90.0	9	81.8	16	84.2
Reads SMS from programme, *n* (%)									
	All	30	75.0	7	70.0	9	81.8	13	68.4
	Most	1	2.5	3	30.0	0	0.0	6.0	31.6
	Some	9	22.5	0	0	2	18.2	0	0
Perceived usefulness of content of SMS messages, *n* (%)									
	Always useful	21	52.5	4	40.0	6	54.5	11	57.9
	Very often useful	18	45.0	6	60.0	5	45.5	7	36.8
	Rarely useful	1	2.5	0	0	0	0	1	5.3
Self‐reported implementation of MCN advice, *n* (%)									
	All or most	24	60.0	8	80.0	7	63.6	9	47.4
	Some	11	27.5	1	10.0	4	36.4	6	31.6
	Almost never	5	12.5	1	10.0	0	0	4	21.0

### 3.2 Refined IPTS and CMO‐Configuration Explain the Divers of Sustained Engagement

Table 3 presents the empirically tested and refined IPTs along with the seven CMOs derived from the data, which validated, extended, and challenged the initial IPTs' assumptions. These CMOs, derived from the empirical data, identify the specific mechanisms that drive sustained engagement with mNutrition among mothers in rural Tanzania. They demonstrate how these mechanisms are activated by mothers' unique contexts—and, in some cases, by experienced inequities within these contexts—to foster and sustain engagement. A narrative explanation of these findings follows (Table 3).

CMO1 highlights that in contexts where mothers experienced high levels of anxiety about their child's health—often rooted in past traumas such as a child's illness or loss—and felt a profound sense of helplessness, especially when access to regular MCHN services was limited, regular, tailored SMS messages with practical MCN advice became a crucial source of reassurance and comfort (Table 3, CMO1). Receiving these messages regularly provided consistent emotional support, helping to alleviate their anxieties. As a result, these mothers sustained their engagement with the programme, valuing the regular reassurance it offered. As 1 s‐time mother explained:

In the past when I gave birth to my first child, I did not know much and made some mistakes when feeding her, as a result she was ill all the time and was only 8 kg or so when she was two. We were so scared that she would die. Now I am so worried all the time that it will be the same with the second child (C1), but these messages help and make me calmer as I know and can do better now (M1).

(21‐year‐old mother, 2 children, poor, Iringa Rural)

CMO2 illustrates that SMS messages were highly valued as they offered much‐needed nurturing emotional support, recognition, and acknowledgement. These messages counteracted feelings of being overworked, undervalued, and unseen within their households (Table 3, CMO2). While the primary purpose of the messages was educational, their tone and personalisation—often addressing mothers directly and beginning with nurturing phrases like ‘beloved mum’—provided an emotional uplift that resonated with mothers, especially those juggling many demanding responsibilities at home. As one overworked mother with three young children explained:

I continue to read the SMS (O2) because I feel special when I open it [the message] and find ‘Dear mama’ (M2). I feel like they always remember me.

(26‐year‐old mother, 3 children, less poor, Mufindi)

CMO3 emerged mainly from empirical data collected from unmarried and young first‐time mothers, who often described feeling lonely, isolated, and disconnected from their previous social networks after having a baby. Societal expectations surrounding motherhood, particularly the expectation to be married, deepened their sense of inadequacy and marginalisation (Table 3, CMO3). These inequities in social support and acceptance were partially mitigated by regular SMS messages, which were framed in an empathetic and supportive tone. These messages provided a sense of connection, offering digital social support and the feeling of belonging to a larger community of new mothers with shared experiences, helping to counteract their isolation.

Most of the time I'm just alone at home and don't talk to anybody. The father of my son has another family and does not come often (C3). The messages are like they have seen my real situation and want to help me. This makes me feel good (M3) and is why I continue reading them (O3).

(17‐year‐old mother, 1 child, poor, Mufindi)

CMO4 shows that mothers remained engaged with the programme because the SMS messages offered authoritative yet supportive guidance on child nutrition and care. Mothers explained that these messages helped them navigate gender inequities and power imbalances in their male‐dominated households more effectively by providing a credible external voice to back their caregiving decisions (Table 3, CMO4). The message lent weight to their arguments during negotiations around childcare and feeding practices, empowering them within their household dynamics. For instance:

I often must spend a lot of time away from the house on the farm because I have to breastfeed the baby often. When I come home my husband was annoyed and shouted at me because I was away so long. I tried to explain but he did not believe me (C4). Now, I show him the messages that say what a mother is supposed to do [with regards to the frequency of breastfeeding]. He now believes and understands the importance of breastfeeding often and I feel good (M4).

(21‐year‐old mother, 2 children, poor, Iringa Rural)

CMO5 highlights a context of inequities related to time poverty, where mothers had access to MCHN services but were deterred from using them due to demanding schedules and multiple responsibilities. This limitation often led to feelings of guilt and inadequacy in their roles as mothers (Table 3, CMO5). In such cases, SMS messages with easily accessible advice were especially welcomed, as they could be read and revisited at a time and place that suited the mothers' busy lives. These messages provided practical support and helped to alleviate feelings of guilt. As a full‐time, out‐of‐home working mother of three children described:

I want to continue to use it [mNutrition] (O5) because the messages help with caring for my children well. I must work all day and miss most seminars at the clinic on how to feed and care for them well. I felt bad about this (C5) but now the messages give me all important information that I need, which makes me feel better (M5).

(26‐year‐old, 3 children, less poor, Mufindi)

CMO6 addresses the inequities stemming from mothers' previous negative experiences with power imbalances in healthcare settings, which discouraged them from seeking MCN advice (Table 3, CMO6). Mothers recounted feeling disrespected or dismissed by overworked healthcare workers when asking for guidance. Young first‐time mothers, in particular, described feeling vulnerable in environments that lacked privacy, where their questions could be overheard by other community members. In this context, anonymous SMS messages framed in a non‐judgmental and supportive tone were highly valued, providing reassurance and reducing the stress associated with seeking advice. An example of a supportively framed *message is:*

Dear mother, do not worry; even a few drops of the first breast milk is enough to satisfy your newborn child. The child's stomach is the size of his/her fist.

(mNutrition message)

A mother described her experience as follows:

I normally afraid to ask the nurse, I am scared she will tell me off (C6). The messages make me feel peaceful (M6), like I am completing a training on how to keep my child well without needing to ask.

(20‐year‐old, 2 children, less poor, Iringa Rural)

In CMO7, mothers perceived the SMS messages as being sent by the government, a perception partly accurate as mNutrition was implemented in partnership with the Government of Tanzania (Table 3, CMO7). This belief led to fears that their childcare behaviours were being monitored by officials. For many mothers, disengagement was not considered an option due to concerns about potential criticism or repercussions. However, the emotional toll of feeling constantly watched began to negatively affect the well‐being of some mothers.

When I get the messages, it feels like somebody is watching me and has seen my real situation. The messages tell me that I must put more effort into feeding the child. They are watching me all the time and might scold me if I don't do as they say.

(43‐year‐old mother, 5 children, poor, Iringa Rural)

While this mechanism effectively sustained engagement, it inadvertently perpetuated power inequities, leveraging fear rather than trust as the driving force behind participation.

The seven CMO configurations offer evidence‐based insights into who, why, and in what contexts mothers sustained their engagement with mNutrition. However, it is important to note that the causal explanations for sustained engagement were rarely driven by a single context or mechanism for individual mothers. Instead, they reflected a complex interplay of multiple contexts and mechanisms. Furthermore, as time passed, mothers often moved through different contexts, each triggering new mechanisms that contributed to their sustained engagement.

## 4 Discussion

Low engagement significantly limits the effectiveness of SMS‐based BCC programmes (Evans et al. 2022). This study directly addressed this limitation by focusing on one underexplored dimension: the mechanisms and contextual conditions that motivate sustained engagement. Using a realist evaluation approach, we examined how the features of the mNutrition programme interacted with mothers' lived realities—particularly structural and social inequities—to trigger engagement over time. The study found that that no single feature of mNutrition alone explained sustained engagement. Rather, engagement was a result of how programme resources—such as message content, tone, and delivery mode—met context‐specific needs rooted in systemic inequities. This has functional implications: digital BCC interventions that only address technical access or information delivery, without considering emotional, social, and structural factors, may fall short in sustaining user interaction. For example, in contexts marked by maternal anxiety or trauma, regular SMS messages functioned as emotional support (CMO1). In settings where women felt socially undervalued or isolated, the tone and framing of messages provided affirmation and a sense of belonging (CMO2, CMO3). These mechanisms reveal that sustained engagement hinges on the ability of digital health programmes to acknowledge and respond to users' psychosocial environments—not just their informational needs. Importantly, some mechanisms, like fear of surveillance (CMO7), sustained engagement through stress rather than trust, underscoring the importance of user‐centred message framing. Programmes that unintentionally leverage coercive perceptions risk exacerbating emotional strain, especially among already marginalised users,. as noted in other studies (Barnett et al. 2022). To enhance equity, it is essential to frame perceived monitoring as supportive rather than punitive, particularly for mothers already facing systemic vulnerabilities.

It is important to highlight that these mechanisms were not universally triggered but were influenced by the interplay between the programme's features and mothers' often inequity‐laden contexts, such as time poverty (CMO5), intrahousehold gender inequities in decision‐making (CMO4), and power imbalances or marginalisation in healthcare settings (CMO6).

While previous literature has discussed the influence of context on engagement with digital BCC, few studies have explicitly examined how users' specific contexts interact with the resources provided by digital BCC programmes to motivate engagement (Baumel et al. 2019; Litterbach et al. 2017; Perski et al. 2017; Redfern et al. 2016; Wong et al. 2010).

Mothers' needs extended beyond credible MCN information to include SMS delivery features (e.g., regularity, flexibility, privacy, and 24‐h access), the tone of messages (non‐judgmental, supportive, and empathetic), and perceptions of the sender (e.g., belief that messages came from trusted authorities). Delivery mode has been identified in prior studies as a key driver of engagement when aligned with users' preferences and contexts (Michie et al. 2017; Perski et al. 2017; Yardley et al. 2016). Message tone has also been linked to engagement in SMS‐based health programmes, though mostly in fostering initial rather than sustained engagement (Habibović et al. 2014; Redfern et al. 2016). Similarly, perceptions of the sender's authority and credibility have been shown to influence SMS programme engagement (Acharya et al. 2023). Findings also suggest that sustained engagement is dynamic and evolves over time. Programmes should incorporate user feedback mechanisms to adapt content, tone, and delivery to changing needs, ensuring continued relevance and engagement.

The absence of structural barriers among sustained engagers suggests that SMS‐based BCC may best serve a relatively privileged subset of the target population. While SMS is often described as equitable due to its low cost and simplicity, this finding supports prior concerns that digital health interventions can reinforce or deepen inequities if not intentionally designed for inclusivity (Michie et al. 2017; Rothstein et al. 2021).

These findings also highlight the limitations of expert‐driven message content design when not paired with participatory input from the local community. Even evidence‐based content, if not co‐developed with end‐users, can risk being misinterpreted—particularly in communities with a history of systemic oversight or marginalisation. Incorporating participatory design processes could help ensure that message tone and framing are culturally resonant, reduce unintended perceptions of surveillance or judgement (see CMO7), and strengthen trust and engagement with digital health programmes.

A key strength of this study is its realist evaluation design, which allowed us to explore not only whether engagement occurred, but why it occurred—and under what conditions. This enabled a deeper understanding of the mechanisms that sustain engagement, grounded in mothers' lived experiences of inequity. The use of theory‐informed, iterative analysis, alongside maximum variation sampling across diverse rural contexts, contributed to the transferability and credibility of the findings. The study also adds a rare focus on sustained engagement, rather than just initial uptake, a critical gap in digital health literature.

A key limitation is that sustained engagement was based on self‐reported data rather than objectively verified mobile phone records, due to data access restrictions. This introduces potential recall bias. The focus on mothers who reported sustained engagement, while valuable for understanding enabling conditions, excluded those who disengaged or never engaged groups whose perspectives are also vital for equitable programme design. Additionally, engagement was captured at a single time point, limiting insights into its evolution over time. Future studies should employ longitudinal methods and include disengaged users to capture a fuller picture of engagement dynamics.

## 5 Conclusion

This study shows that sustained engagement with digital BCC programmes depends not just on message content and delivery, but on how these features respond to users' lived realities—especially structural challenges like time poverty, social isolation, and gendered power dynamics. Using a realist approach, we identified key mechanisms—such as emotional support, social affirmation, and message credibility—that help maintain engagement. Functionally, these insights suggest that effective digital health programmes must: (1) recognise engagement as a dynamic and context‐dependent process; (2) meet users' non‐informational needs such as emotional support and social affirmation; (3) avoid coercive or fear‐inducing messaging; and (4) use realist approaches to tailor design for vulnerable populations most affected by structural inequities. These findings suggest that SMS‐based BCC can serve as more than an information tool—it can be a trusted digital companion for mothers, especially where conventional services are limited.

## Author Contributions

Inka Barnett and Jessica Gordon designed the study and supervised the fieldwork. Deogardius Medardi supervised the fieldwork and preliminary analysis during data collection. Inka Barnett and Jessica Gordon conducted the data analysis. Inka Barnett drafted the initial manuscript. Mieke Snijder reviewed the research design and discussed the results. All authors contributed to the interpretation of the data and the writing of the manuscript and approved the final version.

## Conflicts of Interest

The authors declare no conflicts of interest.

## Data Availability

The quantitative data supporting the findings of this study are openly available in the Harvard Dataverse repository at https://doi.org/10.7910/DVN/NBTDRX. The qualitative data are available on request from the corresponding author to ensure the privacy and confidentiality of the participants.

## Appendix 1 Example mNutrition SMS Sent via the Wazazi Nipendeni SMS Programme.

**Table:**

**Targeted life stage**	**SMS***
Pregnancy	Mom, make sure you eat 3 meals a day and snacks between meals. Eating different types of foods improves your health and helps in the growth of your unborn child.
	You should eat three meals a day and snacks between meals to help the baby to grow properly and so that you have more energy.
	Dear mother, do you know that using folic acid helps in a safe pregnancy and reduces the risks for you and baby during childbirth?
	Do you know that nausea decreases if the iron pill is swallowed up with food or taken at night before bedtime?
When the child is < 6 months of age	Beloved mother, your breast milk for the first 6 months helps a child grow physically and mentally. Giving drinks and other foods to the baby can harm the growth of the child.
	Are you worried that your baby does not get enough milk? If you are unsure, go to the health care provider for advice.
	Mother's milk up to 6 months is enough for a child to grow well and contains enough water to quench the child's thirst.
	When you are away from the baby, put some breast milk in a cup before you leave so that the baby can be fed by somebody else.
When the child is 6–11 months	Dear mother, remember to give your baby fruits and vegetables containing Vitamin A such as pumpkin, carrots, spinach, papaya or mango.
	The mother give the baby food at least four or five times a day while she continues to breastfeed.
	Dear mother, continuing to breastfeed the baby after 6 months contributes to improving nutrition and child health. Mother's milk protects the baby against various diseases.
	It may not be easy for a baby to eat meat. Make sure the meat is crushed so the baby can eat and swallow. The sauce does not have many nutrients unlike meat.
When the child is 12–23 months	Do you have the habit of giving your baby plenty of fatty foods? Most fat contributes to the excess weight of the baby. Do not give your baby a lot of fatty foods!
	Does your child have time to play? Games are very important for children as they prevent them from being overweight. Make sure your baby is playing every day.
	The child should be given three meals a day and snacks, such as fruits, nuts, potato, beans, etc., between meals.
General Maternal and Child (at any time)	Beloved mothers, prepare food for the child in a clean environment to protect the child from diseases and illness.
	Beloved parents do not be afraid. Worm treatments are available free of charge at health care centres. Do not miss this service, it is important for your child!
	It is best to boil drinking water for the child at all times to prevent disease.

*SMS were delivered in Kiswahili; this table includes English translations of SMS provided by Cardno, the company responsible for implementation of *Wazazi Nipendeni*.

## Appendix 2 Initial Programme Theories (IPTs).

**Table:**

**IPT 1:** A mother is more likely to sustain engagement with mNutrition when her child experiences persistent poor health or nutritional challenges, as she perceives a frequent need for the advice provided.
**IPT 2:** Sustained engagement with mNutrition can be triggered when mothers face internal or external barriers and systemic inequities in accessing conventional MCN information sources, provided they have an unmet need for such information.
**IPT 3:** Engagement with mNutrition is influenced by the simplicity and convenience of SMS‐based delivery of MCN information, which accommodates mothers' time constraints and daily routines.
**IPT 4:** A mother's engagement with mNutrition is shaped by her social environment, including intra‐household power dynamics and the level of household support she receives.
**IPT 5:** A mother's perceptions or beliefs about the sender of the SMS‐based programme, whether accurate or not, significantly influence her trust in and engagement with the messages.
